# Gastric Perforation as a Manifestation of Sarcoidosis: A Case Report

**DOI:** 10.1155/crgm/7100284

**Published:** 2026-10-21

**Authors:** Sanjana L. Thimmannagari, John Shrestha, Asim Khanfar, Sungeun Kim, Anthony J. Ocon

**Affiliations:** ^1^ Department of Internal Medicine Rochester General Hospital 1425 Portland Ave Rochester 14621 New York USA rochesterregional.org; ^2^ Department of Pathology and Laboratory Medicine Rochester Regional Health 1425 Portland Ave Rochester 14621 New York USA rochesterregional.org; ^3^ Division of Allergy Immunology and Rheumatology Rochester Regional Health 222 Alexander St Ste 300 Rochester 14607 New York USA rochesterregional.org

## Abstract

**Background:**

Gastrointestinal involvement in sarcoidosis is rare, and clinically apparent disease occurs in fewer than 1% of patients. The stomach is the most frequently affected site, although presentations are usually indolent. Perforation is exceedingly rare, and to our knowledge, this is the first case of gastric perforation attributable to sarcoidosis.

**Case Presentation:**

A 30‐year‐old woman presented with 4 months of bilateral lower extremity paresthesia, pleuritic chest pain, progressive dyspnea, abdominal discomfort, and 50 lb unintentional weight loss. Angiotensin‐converting enzyme and soluble interleukin‐2 receptor were markedly elevated, and autoimmune serologies and extensive infectious workup were negative. Imaging revealed abdominal and pelvic lymphadenopathy, hepatosplenomegaly, and bilateral cavitary pulmonary opacities with mediastinal and hilar lymphadenopathy.

During admission, she developed acute abdominal pain with pneumoperitoneum. Emergent laparotomy revealed a 6‐mm gastric perforation repaired with a Graham patch, with biopsies revealing lymphohistiocytic aggregates and multinucleated giant cells containing asteroid bodies. Subsequent endobronchial ultrasound–guided mediastinal node biopsy demonstrated granulomatous lymphadenitis, and a bronchoalveolar lavage showed a cD4/CD8 ratio of 15:1, suggesting sarcoidosis. Glucocorticoids were deferred due to surgical repair initially, and hydroxychloroquine was started. Subsequent lung wedge resection confirmed non‐necrotizing granulomas. Mycophenolate mofetil was then added and replaced by methotrexate with clinical improvement and resolution of symptoms.

**Conclusion:**

Gastric perforation can be a severe complication in sarcoidosis, particularly in patients with multisystemic symptoms that are not otherwise explained. Multidisciplinary involvement is needed to appropriately treat these patients.

## 1. Introduction

Sarcoidosis is a multisystemic disorder of unknown etiology typically characterized by the presence of noncaseating granulomas classically involving the lungs and intrathoracic lymph nodes but may affect any organ of the body [1, 2].

Involvement of the hollow viscus of the gastrointestinal (GI) tract is a rare manifestation of sarcoidosis and is reported in approximately 0.1%–1.6% of cases, with clinically apparent symptoms present in fewer than 1% of patients [3]. When the GI lumen is affected, the distribution is uneven and follows an oral‐anal gradient [4]. GI manifestations may present in isolation, most frequently involving the antrum of the stomach [5]. Gastric sarcoidosis may present as mucosal ulceration, nodules, or polypoidal lesions leading to epigastric pain, weight loss, nausea, vomiting, bleeding, and even gastric outlet obstruction [1, 6]. Although most diseases are benign, some cases may have severe complications such as GI bleeding or obstruction [5]. However, the small intestine is usually spared, a feature that helps distinguish it from Crohn’s disease [5].

To our knowledge, this is the first reported case of gastric perforation attributed to sarcoidosis.

## 2. Case Presentation

A 30‐year‐old female presented with a 4‐month history of intermittent abdominal discomfort, pleuritic chest pain, progressive dyspnea, occasional small‐volume hemoptysis, nonproductive cough and bilateral lower extremity tingling and numbness. She also endorsed amenorrhea, nausea, vomiting, anorexia, fever, and chills. In addition, she reported unintentional weight loss of approximately 50 pounds from her baseline weight of 190 pounds over the past few months.

Her past medical history was only notable for bacterial vaginosis. She denied recent travel or sick contacts. She was employed as a medical technician. She denied use of alcohol, tobacco, or intravenous drugs, and had prior marijuana use. She had no history of incarceration, homelessness, pet ownership, or significant outdoor or chemical exposure.

On presentation, she was febrile to 101.7°F and tachycardic with a heart rate in the 120s. Physical examination revealed pallor, normal bilateral vesicular breath sounds, normal but rapid heart sounds, abdominal distention with diffuse tenderness most prominent in the right upper quadrant, and nontender inguinal lymphadenopathy. Weight was 140 lbs.

Hematologic studies demonstrated anemia with hemoglobin 9.3 g/dL with normal leukocyte and platelet counts. Chemistry was notable for hyponatremia with serum sodium of 129, but remaining electrolyte levels, including calcium, were within range. Liver function tests were notable for elevated aspartate aminotransferase (AST) of 96 U/L and alkaline phosphatase (AP) levels of 831 U/L. Autoimmune serologies, including antinuclear antibody, rheumatoid factor, anti‐Jo‐1 antibody, and antineutrophil cytoplasmic antibodies (ANCAs), were negative, with normal creatine kinase and aldolase levels. Additional tests found elevations in C‐reactive protein (CRP) of 71.3 mg/L, erythrocyte sedimentation rate (ESR) of 28 mm/hr, lactate dehydrogenase (LDH) of 1016 U/L, ferritin of 3990 ng/mL, angiotensin‐converting enzyme (ACE) level of 266 U/L, and soluble IL‐2 receptor level of 6675.6 U/mL. Given the multisystem presentation, constitutional symptoms, lymphadenopathy, and marked laboratory abnormalities, the initial differential diagnosis remained broad and included disseminated infection, malignancy, and systemic inflammatory or autoimmune disease.

Initial CT of the abdomen and pelvis revealed extensive abdominal and pelvic lymphadenopathy as well as hepatosplenomegaly. Given history of dyspnea, pleuritic chest pain, and hemoptysis, a CT angiogram of the chest was pursued, which was negative for pulmonary embolism but revealed bilateral alveolar opacities with cavitation, along with reactive mediastinal and bilateral hilar lymphadenopathy (Figure 1).

**FIGURE 1:**
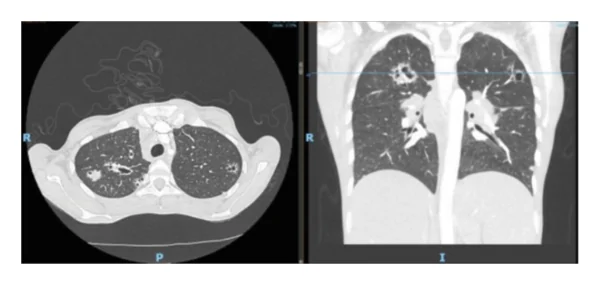
CT chest imagery. Cross‐sectional (left) and coronal (right) planes of CT chest revealing bilateral cavitary lesions and hilar lymphadenopathy.

Infectious disease was consulted early in the diagnostic evaluation because of concern for a disseminated infectious process. An extensive microbiological workup, including HIV testing, blood cultures, acid‐fast bacilli smears, QuantiFERON testing for tuberculosis, beta‐D‐glucan, histoplasma testing, cytomegalovirus, Epstein–Barr virus, and *Aspergillus* testing, was unrevealing.

During hospitalization, the patient developed sudden acute worsening of abdominal pain, and a repeat CT of the abdomen and pelvis revealed new pneumoperitoneum (Figures 2a and b). She underwent emergent exploratory laparotomy, which found a 6‐mm perforation of the anterior gastric wall, successfully repaired with a Graham patch. Full‐thickness biopsy of the gastric wall at the edge of the ulcer intraoperatively revealed scattered lymphohistiocytic aggregates with multinucleated giant cells containing asteroid bodies (Figures 3a andb). These histopathologic findings raised concern for an underlying granulomatous disorder.

**FIGURE 2:**
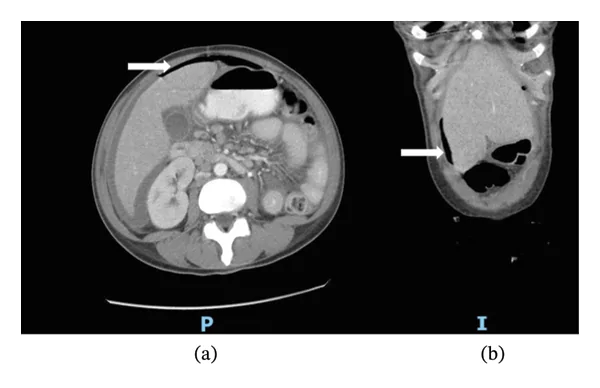
CT of the abdomen and pelvis. Cross‐sectional (a) and coronal (b) planes revealing pneumoperitoneum (arrow).

**FIGURE 3:**
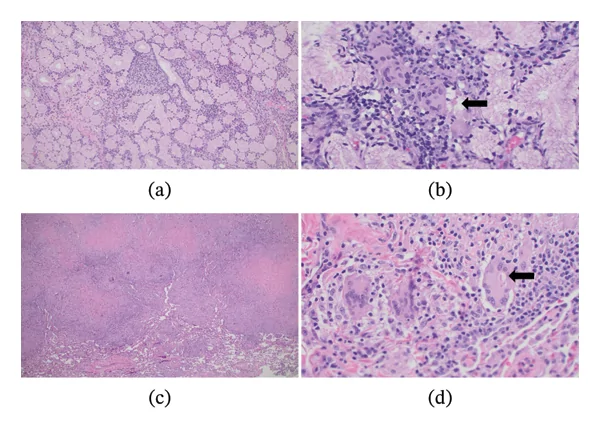
Histopathology of gastric and pulmonary biopsy specimens: (a) gastric mucosa with a focus of lymphohistiocytic aggregate and multinucleated giant cells, (b) asteroid body (arrow) within a giant cell in gastric mucosa, (c) multiple non‐necrotizing granulomas in the lung parenchyma, and (d) multinucleated giant cells containing asteroid body (arrow) within granulomas of the lung parenchyma.

Given evidence of extensive lymphadenopathy and hepatosplenomegaly with elevated LDH, there was concern for underlying malignancy, and hematology was also consulted. Postoperatively, pulmonology was consulted, and she underwent a bronchoscopy with bronchoalveolar lavage (BAL) and endobronchial ultrasound (EBUS)–guided fine‐needle aspiration (FNA) of a mediastinal lymph node, with histology demonstrating granulomatous lymphadenitis. There was no evidence of aberrant T‐ or B‐cell populations on flow cytometry. Flow cytometry of BAL fluid revealed a markedly elevated CD4/CD8 ratio of 15:1.

The combination of granulomatous inflammation in the mediastinal lymph node and gastric tissue, bilateral hilar and mediastinal lymphadenopathy, cavitary pulmonary lesions, elevated ACE and soluble interleukin‐2 receptor levels, and a markedly elevated BAL CD4/CD8 ratio, together with an extensive negative infectious workup, suggested a diagnosis of systemic sarcoidosis with pulmonary and GI involvement.

Rheumatology was consulted to guide immunosuppressive management. The recent gastric perforation and surgical repair posed a therapeutic challenge because of concern for impaired postoperative wound healing with early glucocorticoid therapy. Following multidisciplinary discussion, glucocorticoid therapy was initially deferred. Hydroxychloroquine was started as initial therapy, and corticosteroids were subsequently initiated 10 days postoperatively once the immediate postoperative period had passed.

She re‐presented to the hospital 1 month later with chest pain and dyspnea. CT of the chest demonstrated a new left‐sided pleural effusion and collapse of the left lower lobe, in addition to previously noted cavitary lesions. Thoracentesis yielded an exudative pleural effusion, with microbiological and cytological studies negative for infection and malignancy. Given the persistent pulmonary abnormalities and pleural involvement, she subsequently underwent video‐assisted thoracoscopic surgery with wedge resection of the lung. Histopathologic examination demonstrated non‐necrotizing granulomas with multinucleated giant cells, providing additional tissue confirmation of pulmonary sarcoidosis (Figures 3c and d). Mycophenolate mofetil was subsequently added to her regimen as a steroid‐sparing agent. Due to persistence of symptoms, she was eventually transitioned to methotrexate. The patient is currently doing well, with normalization of her previously elevated inflammatory markers, soluble interleukin‐2 receptor, and ACE levels. She is currently stable on methotrexate 2 years out, has regained her weight, and did not report any symptoms at her last pulmonology appointment. Although her paresthesias were not formally worked up inpatient due to her more critical condition at the time, her paresthesias improved by the time she was discharged from the hospital, and she attributed the improvement in paresthesias to starting immunosuppressive therapy. Therefore, it is assumed, but cannot be confirmed, that her paresthesias were related to sarcoidosis. Given resolution, outpatient nerve conduction testing was deferred.

## 3. Discussion

While pulmonary sarcoidosis is the most prevalent clinical presentation of sarcoidosis, extrapulmonary manifestations including abdominal involvement are well recognized. However, luminal GI involvement remains rare [1]. Although it is uncommon, luminal GI involvement can be clinically significant, with life‐threatening presentations seen in up to 22% of cases [4]. Sarcoidosis may also present elsewhere in the intra‐abdominal cavity and can mimic malignancy due to nonspecific lymphadenopathy on imaging [7, 8].

Within the GI tract, the disease process follows an oral‐anal gradient with around 80% of cases involving the esophagus, stomach, and duodenum [4]. Gastric involvement is detectable in only 10% of patients and is usually asymptomatic. However, when gastric involvement is present, clinical manifestations may include weight loss, dyspepsia, satiety, bloating, anemia, and, in 10% of cases, upper GI bleeding [1]. In a review of 305 cases of GI sarcoidosis, gastric sarcoidosis was reported to present as diffuse gastric infiltration mimicking malignancy (58%), ulcerative disease and polypoidal lesions [4]. While prior reports have described esophageal and appendiceal perforation [9, 10], gastric wall perforation as in the presented case is rare. While major reviews list perforation as one of the life‐threatening complications of GI sarcoidosis, no prior gastric perforation attributed to granulomatous infiltration due to sarcoidosis has been reported to date.

One of the diagnostic challenges in this case, and a limitation, was the absence of well‐formed noncaseating granulomas in the gastric specimen, and biopsy specimens demonstrated a lymphohistiocytic aggregate and multinucleated giant cells, containing an asteroid body. This finding required a critical clinical interpretation given that the histopathological hallmark of sarcoidosis is noncaseating granulomatous inflammation formed by a core of epithelioid histiocytes and multinucleated giant cells with sparse lymphocytes [1, 2, 11]. Sarcoid giant cells may harbor cytoplasmic inclusions such as asteroid bodies, Schaumann bodies, and Hamazaki–Wessberg bodies, which are nonspecific as well as nonpathognomonic [12]. The rarity of formed granulomas in GI sarcoidosis is expected and is clinically meaningful too. In the largest endoscopic histologic series, only 1 out of 230 patients with a diagnosis of sarcoidosis who underwent GI biopsies had a noncaseating granuloma [13]. The authors here concluded there may be a low probability of finding characteristic histology in GI biopsy.

Identifying an asteroid body within the giant cells in gastric biopsy provided a histologic continuity with the biopsies from her mediastinal lymph node and wedge resection specimens, which demonstrated noncaseating granuloma with giant cells containing asteroid bodies, and supported a diagnosis of sarcoidosis, particularly in the absence of any other identified etiologies.

Since there is no single confirmatory test for diagnosis of sarcoidosis, the American Thoracic Society has set forward three pillars for diagnosis, including a compatible clinical and radiologic presentation, demonstration of noncaseating granuloma in one or more tissues and exclusion of alternative causes of granulomatous disease [11]. The criteria are applied to extra‐thoracic involvement as well, and it is quite important to rule out mimics in GI disease. The constellation of signs, including non‐necrotizing granulomas in pulmonary tissues, multiorgan involvement, and exclusion of other causes of granulomatous disease, further solidified the diagnosis of gastric sarcoidosis.

Acute gastric perforation, as seen in our case, requires emergent surgical repair. Beyond the acute intervention, glucocorticoids remain an effective treatment agent. Several reports have documented prompt symptomatic improvement with high‐dose corticosteroid therapy [14, 15]. Steroid‐sparing immunosuppressants, such as methotrexate and mycophenolate mofetil, may also be effective adjuncts [1, 4]. For refractory cases, TNF inhibitors, particularly infliximab and adalimumab, have been shown to be efficacious in the treatment of both pulmonary and extrapulmonary disease [16].

In conclusion, GI involvement in sarcoidosis remains underrecognized, with various presentations ranging from asymptomatic to gastric perforation as exemplified by this case. Given its significant implications in patients’ morbidity, a high degree of clinical suspicion for GI involvement is necessary when a patient with sarcoidosis presents with acute abdominal pain.

## Author Contributions

Sanjana L. Thimmannagari and John Shrestha prepared the initial draft of the manuscript. Asim Khanfar, Sungeun Kim, and Anthony J. Ocon revised the manuscript. All authors reviewed the manuscript.

## Funding

No funding was received for the development of this study.

## Consent

Informed consent was obtained from the patient, and potential identifiers have been omitted and anonymized according to the International Committee of Medical Journal Editors (ICMJE) recommendations.

## Conflicts of Interest

Anthony Ocon MD PhD is a consultant and speaker for Amgen Inc., independent of this work. The remaining authors declare no conflicts of interest.

## Data Availability

Data sharing is not applicable as no new data were generated.

## References

[1] Nicolosi S. , Chernovsky M. , Angoni D. et al., Gastrointestinal Manifestations of Sarcoidosis: A State-of-the-Art, Comprehensive Review of the Literature—Practical Clinical Insights and Many Unmet Needs on Diagnosis and Treatment, Pharmaceuticals. (2024) 17, no. 9, 10.3390/ph17091106. PMC11435091 39338270

[2] Polverino F. , Balestro E. , and Spagnolo P. , Clinical Presentations, Pathogenesis, and Therapy of Sarcoidosis: State of the Art, Journal of Clinical Medicine. (2020) 9, no. 8, 10.3390/jcm9082363. PMC7465477 32722050

[3] Brown I. and Kumarasinghe M. P. , Granulomas in the Gastrointestinal Tract: Deciphering the Pandora’s Box, Virchows Archiv. (2018) 472, no. 1, 3–14, 10.1007/s00428-017-2210-3. 28776106

[4] Brito-Zerón P. , Bari K. , Baughman R. P. , and Ramos-Casals M. , Sarcoidosis Involving the Gastrointestinal Tract: Diagnostic and Therapeutic Management, Official journal of the American College of Gastroenterology | ACG. (2019) 114, no. 8, 1247, https://journals.lww.com/ajg/fulltext/2019/08000/sarcoidosis_involving_the_gastrointestinal_tract_.14.aspx, 10.14309/ajg.0000000000000171. 30865014

[5] Eklund A. and du Bois R. M. , Approaches to the Treatment of Some of the Troublesome Manifestations of Sarcoidosis, Journal of Internal Medicine. (2014) 275, no. 4, 335–349, 10.1111/joim.12198. 24433397

[6] El Sharu H. , Ibarra S. , Chaudhary A. , Hidri S. , Khan Z. , and Hoo-Fatt D. , Gastric Sarcoidosis Diagnosed With Endoscopic Ultrasound, Clinical Case Reports. (2024) 12, no. 3, 10.1002/ccr3.8623. PMC10932793 38481933

[7] Bulut Gokten D. and Mercan R. , Gastrointestinal Sarcoidosis Mimicking Malignancy: A Confusing Scenario, Caspian Journal of Internal Medicine. (2025) 16, no. 3, 584 EP–587, 10.22088/cjim.16.3.584. PMC12329357 40786581

[8] Bains L. , Paswan N. , Khan S. A. , and Mandal S. , Abdominal Sarcoidosis: A Mystery Revisited, Clinical Case Reports. (2025) 13, no. 12, 10.1002/ccr3.71557. PMC12689946 41383541

[9] Rustagi T. and Majumder S. , Dysphagia and Spontaneous Esophageal Perforation in Sarcoidosis, Digestive Diseases and Sciences. (2013) 58, no. 1, 282–285, 10.1007/s10620-012-2336-8. 22923314

[10] Munt P. W. , Sarcoidosis of the Appendix Presenting as Appendiceal Perforation and Abscess, Chest. (1974) 66, no. 3, 295–297, 10.1378/chest.66.3.295. 4422824

[11] Crouser E. D. , Maier L. A. , Wilson K. C. et al., Diagnosis and Detection of Sarcoidosis. An Official American Thoracic Society Clinical Practice Guideline, American Journal of Respiratory and Critical Care Medicine. (2020) 201, no. 8, e26–e51, 10.1164/rccm.202002-0251ST. PMC7159433 32293205

[12] Belperio J. A. , Shaikh F. , Abtin F. G. et al., Diagnosis and Treatment of Pulmonary Sarcoidosis: A Review, JAMA. (2022) 327, no. 9, 856–867, 10.1001/jama.2022.1570. 35230389

[13] Wu H. and Shen B. , Endoscopic and Histologic Evaluation of the Gastrointestinal Tract in Patients With Sarcoidosis, European Journal of Gastroenterology and Hepatology. (2020) 33, no. 5, 639–644, 10.1097/MEG.0000000000001943. 33079782

[14] Stampfl D. A. , Grimm I. S. , Barbot D. J. , Rosato F. E. , and Gordon S. J. , Sarcoidosis Causing Duodenal Obstruction, Digestive Diseases and Sciences. (1990) 35, no. 4, 526–532, 10.1007/BF01536930. 2180656

[15] Yousef J. , Loharia J. , Marei T. , and Alarfaj A. , Gastrointestinal Sarcoidosis With Esophageal Involvement-A Rare Clinical Experience, Scholars Journal of Medical Case Reports. (2018) 6, no. 9, 686–689.

[16] Rezaee M. , Zangiabadian M. , Soheili A. et al., Role of Anti-Tumor Necrosis Factor-Alpha Agents in Treatment of Sarcoidosis: A Meta-Analysis, European Journal of Internal Medicine. (2023) 109, 42–49, 10.1016/j.ejim.2022.12.003. 36526497

